# Application of Photo-Fenton Process to Highly Saline Water Matrices: Effect of Inorganic Ions on Iron Speciation

**DOI:** 10.3390/molecules31010056

**Published:** 2025-12-23

**Authors:** Ivan Vallés, Javier Moreno-Andrés, Iván Sciscenko, Lucas Santos-Juanes, Antonio Arques

**Affiliations:** 1 Grupo de Procesos de Oxidación Avanzada, Departamento de Ingeniería Textil y Papelera, Universitat Politècnica de València, Campus de Alcoy, 03801 Alcoy, Spain; ivvalfer@epsa.upv.es (I.V.); ivanmatias.sciscenko@unito.it (I.S.); lusanju1@txp.upv.es (L.S.-J.); 2 Department of Environmental Technologies, Faculty of Marine and Environmental Sciences, INMAR-Marine Research Institute, CEIMAR-International Campus of Excellence of the Sea, University of Cadiz, Campus Universitario de Puerto Real, 11510 Cádiz, Spain; javier.moreno@uca.es; 3 Department of Chemistry, University of Turin, Via Pietro Giuria 5, 10125 Turin, Italy

**Keywords:** iron complexation, radical scavenging, inorganic anions, pollutant degradation, disinfection

## Abstract

The photo-Fenton process has been widely studied for the treatment of organic pollutants and disinfection in a wide range of scenarios. Nevertheless, its efficiency decreased when applied to complex matrices, as in the case of most advanced oxidation processes. Despite the interferences caused by different anions, the photo-Fenton is able to obtain good degradation values for pollutants and microorganisms, especially in combination with other methods; however, it depends on the matrix to be treated. Due to the lack of studies and reviews in this field, this paper reviewed the outcome of the inorganic ions present on highly saline water matrices (more than 1 g L^−1^ of chlorides, fluorides, bromides, sulphates, carbonates or bicarbonates, borates, phosphates and nitrates/nitrites) on the Fenton-based processes, focusing on their outcome on iron speciation and their scavenger effect. Also, the most relevant works so far for the abatement of microcontaminants and disinfection by this process on highly saline matrices have been revised. Special emphasis is on the efficiency of the process, considering the relevant industries referred to.

## 1. Introduction

Advanced oxidation processes (AOPs) have gained considerable attention for wastewater remediation, and among them, Fenton and related treatments stand out due to their operational simplicity and their ability to degrade recalcitrant pollutants [1]. Although the fundamentals of the Fenton reaction have been extensively described, some mechanistic aspects of Fenton chemistry are still under discussion, and alternative pathways have been proposed [2,3,4]. Nevertheless, it is widely accepted that Equations (1) and (2) govern the catalytic cycle [5]:

Fe^2+^ + H_2_O_2_ → Fe^3+^ + HO^−^ + HO^•^ (1)

Fe^3+^ + H_2_O_2_ → Fe^2+^ + HO_2_^•^ + H^+^ (2)

Because Equation (2) is approximately five orders of magnitude slower than Equation (1), Fe(III) reduction becomes the rate-limiting step of the process [6]. Under irradiation (λ < 530 nm), however, Fe(III) photoreduction is accelerated by ligand-to-metal charge transfer (LMCT), as shown in Equation (3) [7]. This photochemical enhancement defines the photo-Fenton reaction, whose efficiency strongly depends on the concentration of Fe(OH)^2+^, maximised at pH 2.8 [8], typically considered the optimal pH for this process [9].

Fe(OH)^2+^ + hν → Fe^2+^ + HO^•^ (3)

As pH deviates from 2.8, the relative abundance of Fe(OH)^2+^ decreases sharply and photo-Fenton efficiency drops accordingly. Above pH 4, Fe(III) rapidly precipitates as (oxo)hydroxides, interrupting the catalytic cycle and suppressing ROS production [10]. This well-known limitation poses significant constraints for large-scale applications, since intensive pH adjustment increases operational costs and generates substantial quantities of iron sludge. The challenge becomes even more pronounced in real waters, where natural alkalinity—particularly the CO_2_(aq)/HCO_3_^−^ buffer system—requires substantial acid consumption to maintain acidic conditions [11]. As a result, extending the effective pH range of the photo-Fenton process remains a major research target.

One strategy to overcome this drawback involves modifying Fe(III) speciation through complexation with anions, dissolved organic matter, or intentionally added chelating agents such as EDDS. In these cases, Fe(III)–ligand complexes (FeL_n_^3−n^) may undergo LMCT according to Equation (4), potentially enhancing or inhibiting Fe(III) photoreduction depending on the ligand nature and stability [12,13,14]:

FeL_n_^3−n^ + hν → Fe^2+^ + nL^+1^ (4)

The presence of inorganic anions in particular produces a strong influence on photo-Fenton performance. These ions can alter the coordination environment of iron, modify Fe(II) regeneration rates and, in some cases, affect the identity and yields of reactive oxygen species (ROS). Their effects, however, are highly ion-specific and often depend on pH and matrix composition. Phosphates, for example, form poorly soluble iron phosphate (FePO_4_, log Ksp = −16 [15]), completely suppressing Fenton reactivity. Nitrates and sulphates, in contrast, show negligible interaction with the process even at high concentrations (up to 1 g L^−1^) [16]. Chlorides have traditionally been associated with inhibitory effects through ROS scavenging [17], although beneficial behaviour has also been reported at near-neutral pH due to changes in iron speciation and enhanced Fe(III) solubility [18]. Beyond speciation, many inorganic ions may act as scavengers of HO•, HO_2_^•^ or ^1^O_2_, altering the oxidative pathways available. The influence of common anions—including nitrates, sulphates, phosphates, carbonates and chlorides—on AOPs and particularly on photo-Fenton chemistry has thus been extensively documented [19,20,21]. Throughout this review, highly saline matrices are considered those containing anion levels typically exceeding 1 g·L^−1^, recognising that the resulting effects on iron speciation and radical chemistry depend strongly on the specific identity of each ion.

In contrast to anions, cations generally do not exert inhibitory effects in photo-Fenton systems. Alkali and alkaline-earth metals (Na^+^, K^+^, Mg^2+^, Ca^2+^) typically show negligible impact [16]. Some transition metals (Cu^2+^, Co^2+^, Cr^3+^) may drive Fenton-like reactions [22], though they are rarely present at significant levels in natural matrices except in specific industrial effluents [23] and are therefore usually added deliberately in engineered treatments [24,25]. For these reasons, their contribution falls outside the scope of this review.

Alongside homogeneous systems, substantial effort has been directed toward the development of supported and heterogeneous photo-Fenton catalysts. Iron species immobilised on carbonaceous substrates, clays, metal oxides or polymeric supports can reduce iron leaching, improve catalyst stability and broaden the operational pH window through surface-mediated LMCT pathways. Recent advances in iron-loaded porous frameworks and hybrid materials have demonstrated promising performance under both UV and visible light, even in saline matrices where iron precipitation is typically a major bottleneck [26,27]. These developments reflect the broader trend of designing photoactive materials capable of operating efficiently in complex water matrices.

In line with these advances, recent studies have explored hybrid photoelectrocatalytic systems that combine carbonaceous supports with iron-doped semiconductor materials to enhance oxidative performance under complex water conditions. For example, Fe-doped porous carbon nitride immobilised on hydrophobic carbon felt has demonstrated highly efficient degradation of organic pollutants through the direct activation of molecular oxygen under irradiation. This type of architecture illustrates how tailored interfaces between iron species, conductive carbon materials and photoactive semiconductors can facilitate alternative ROS-generation pathways beyond classical Fe(III) photoreduction, potentially mitigating the limitations imposed by high salinity and restricted Fe(III) solubility [28].

The application of the photo-Fenton process to real saline effluents also introduces additional considerations related to environmental sustainability and process scalability. High salinity affects solution conductivity, reagent consumption, iron solubility and sludge generation—all factors that influence the viability of pilot- or full-scale implementation. Common saline wastewaters—such as produced water, textile brines or desalination concentrates—often require pH adjustment and high oxidant doses, increasing operational and environmental costs. As a result, recent research has focused on solar-driven systems, low-cost complexing agents enabling circumneutral pH operation, hybrid AOP–membrane treatments, and strategies for brine valorisation or selective reuse [29]. These approaches highlight the need for a comprehensive understanding of how saline matrices modify photo-Fenton reactivity and process sustainability.

In this context, the aim of this review is to systematically analyse the influence of inorganic anions on the performance of photo-Fenton processes. We first discuss mechanistic aspects, including changes in iron speciation, hydroxyl radical quenching and the generation of alternative reactive species. We then examine the specific roles of the most relevant ions and their ion-dependent effects. Finally, we evaluate matrix influences in highly saline industrial effluents and seawater, considering the implications for both pollutant removal and disinfection applications.

## 2. Mechanistic Aspects of Anions on Fenton Process: HO^•^ Quenching or Changes in Iron Speciation?

Changes in the performance of the photo-Fenton process have been comprehensively revised in the presence of anions. In general, anions decrease the availability of reactive species (·OH), thus inhibiting photo-Fenton at different extents. This can be attributed to different factors, the most important of which are the following: (i) the modification of the coordination sphere of iron which changes the Fenton mechanism and, in some cases, the nature of the reactive species (see Equation (4), above), and (ii) the scavenging role of the anion for ·OH (Equation (5), where X^−^ represents the anion).

HO^•^ + X^−^ → OH^−^ + X^•^ (5)

**Figure 1:**
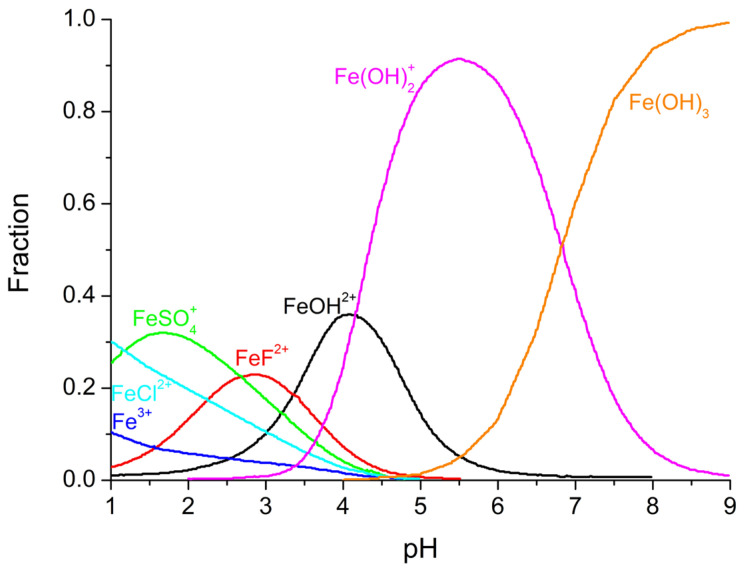
The speciation of Fe(III) in seawater as a function of pH at 25 °C. Adapted from F. Millero [37].

In order to discuss the nature of the interference of each of the most relevant anionic water constituents, it is interesting to consider the reaction rate of ·OH with selected anions (Table 1) and also the ability of these species to complex iron, which is provided in Table 2 and Figure 1.

The contribution of each inorganic ion to HO^•^ quenching varies significantly and strongly depends on pH, ionic strength and radical distribution. Chloride, bicarbonate/carbonate and bromide exhibit the highest scavenging capacities, rapidly converting HO^•^ into less reactive species such as CO_3_^•−^ or Br^•^. These alternative radicals display lower redox potentials and more selective reactivity, thereby decreasing mineralisation rates despite sometimes maintaining pollutant degradation rates. Chloride interaction with hydroxyl radicals is important at acidid pH values; meanwhile, the formation of less active complexes at near-neutral pHs values becomes predominant. In contrast, sulphate and phosphate show moderate HO^•^ quenching effects but exert stronger influence through iron complexation or precipitation [30]. Understanding the balance between radical scavenging and iron speciation is therefore critical to interpret the observed behaviour in complex saline environments.

**Table 1:** Rate constants for the reaction of hydroxyl radicals with relevant inorganic anions according to the literature (references given in the last column).

Reaction	k (M^−1^ s^−1^)	pH	Reference
·OH + Cl^−^ → ClOH^·−^	4.3 × 10^9^	pH = 2	[31]
	1 × 10^3^	pH = 7	[32]
·OH + Br^−^ → BrOH^·−^	1.1 × 10^10^	pH = 1	[31]
·OH + I^−^ → IOH^·−^	1.1 × 10^10^	Not reported	[31]
·OH + F^−^	-		
·OH + HSO_4_^−^ → SO_4_^·−^ + H_2_O	6.9 × 10^5^	pH = 1	[31]
·OH + SO_4_^2−^ → SO_4_^·−^ + OH^−^	Not reported		
·OH + HNO_3_ → NO_3_^·^ + H_2_O	1.4 × 10^8^		[33]
·OH + NO_2_^−^ → NO_2_^·^ + OH^−^	1.0 × 10^10^		[31]
·OH + HPO_4_^2−^ → HPO_4_^·^ + OH^−^	1.5 × 10^5^		[31]
·OH + H_2_PO_4_^2−^ → H_2_PO_4_^·^ + OH^−^	2 × 10^4^		[31]
·OH + HCO_3_^−^ → CO_3_^·−^ + H_2_O	8.5 × 10^6^	pH = 7–9	[31]
·OH + CO_3_^2−^ → CO_3_^·−^ + OH^−^	3.9 × 10^8^	Basic pH	[31]
·OH + H_3_BO_3_ → H_2_O + H_2_BO_3_^·^	5 × 10^4^	pH = 7.3	[31]

**Table 2:** Iron speciation in the presence of relevant inorganic anions and stability constants reported in the literature (references given in the last column). Most data refer to an ionic strength of 0.68 M, which is the standardised value for seawater.

Formed Specie	logβ	Ionic Strength (M)	Reference
Fe(OH)^2+^	−2.71	0.68	[34]
Fe(OH)_2_^+^	−7.07	0.68	
Fe(OH)_3(aq)_	−13.6	0.68	
Fe_2_(OH)_2_^4+^	−2.90	0.5	
FeCl^2+^	0.5	0.6	[35]
FeCl_2_^+^	0.63		
FeF^2+^	5.24	0.7	[36]
FeF_2_^+^	9.46		
FeF_3(aq)_	11.84		
FeH_2_PO_4_^2+^	3.4	0.4	[37]
FeHPO_4_^+^	8.4		
FeSO_4_^+^	2.12	0.6	[38]
Fe(SO_4_)_2_^−^	3.03		
FeCO_3(aq)_	3.65	0.7	[36]
Fe(CO_3_)_3_^−3^	5.19		
Fe[B(OH)_4_]^2+^	6	0.68	[39,40]

The elucidation of degradation pathways in saline matrices can be improved by incorporating selective radical- and electron-trapping experiments. Common scavengers such as tert-butanol or methanol (HO^•^), p-benzoquinone (O_2_^•−^), furfuryl alcohol (^1^O_2_), or specific spin traps for ESR such as DMPO or TEMP, provide valuable information on the contribution of different reactive species. However, in hypersaline systems, competition between trapping agents and inorganic anions can complicate interpretation. For instance, Cl^−^ or HCO_3_^−^ may react faster than the scavenger, masking HO^•^ formation. Future studies should therefore combine multiple probes, kinetic modelling and controlled synthetic matrices to accurately discriminate the formation and fate of HO^•^, CO_3_^•−^, SO_4_^•−^ and halogen radicals in photo-Fenton systems [41].

### 2.1. Chlorides

The effect of chlorides on the Fenton system is probably the one that has deserved more attention from researchers, together with carbonates/bicarbonates. Although Cl^−^ is a weak iron ligand and a low-moderate HO· scavenger at pH 7 (Table 1), it can be found at a high concentration in relevant water matrices; for instance, 30 to 37 g L^−1^ of NaCl can be found in marine water [42]. Equations (6)–(10) are key to describe the effect of Cl^−^ vs. photo-Fenton [43]. Despite the formation of FeCl^+^ possibly occurring (Equation (6)), it does not have a remarkable effect on the first step of the Fenton process (Equation (7)), and the oxidation of Fe(II) into Fe(III) occurs, generating ·OH. On the contrary, the formation of Fe(III) chlorocomplexes (Equation (8)) requires the photoreduction of Fe(III) through (Equation (9)), which is inefficient and gives Cl·, which in turn forms Cl_2_^·−^ (Equation (10)).

Fe^2+^ + Cl^−^ ⇌ FeCl^+^ (6)

FeCl^+^ + H_2_O_2_ → Fe^3+^ + HO^•^ + OH^−^ + Cl^−^ (7)

Fe^3+^ + Cl^−^ ⇌ FeCl^2+^ (8)

FeCl^2+^ + hν → Fe^2+^ + Cl^•^ (9)

Cl· + Cl^−^ → Cl_2_^•−^ (10)

On the other hand, despite the fact that Cl^●^ and Cl_2_^●−^ are thermodynamically strong oxidants (E^○^(Cl^●^/Cl^−^) = 2.41 V and E^○^(Cl_2_^●−^/2Cl^−^) = 2.09 V) [44], they are utterly less reactive and more selective than HO^●^, eventually exhibiting a higher concentration than HO^●^ (due to its accumulation by its low reactivity). Its reactivity with organic compounds can vary from high with phenols to low with aliphatic alcohols [45].

**Figure 2:**
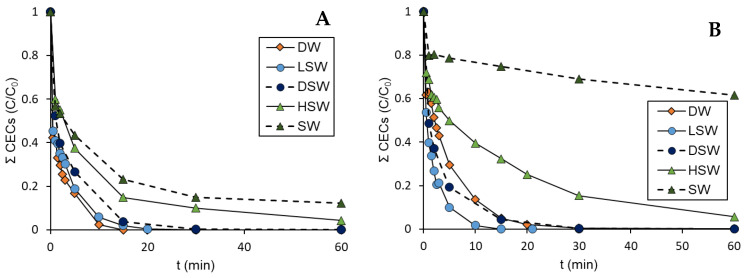
Photo-Fenton treatment of a mixture of pollutants at pH 2.8 (**A**) and pH 5 (**B**) in different aqueous matrices: Distilled Water (DW), Diluted Seawater (DSW), Low salty water (LSW, 1 g L^−1^ NaCl), Seawater (SW) and High salty water (HSW, 30 g L^−1^ NaCl). Graph of ΣCEC/ΣCEC_0_ vs. time. [Fe(II)] = 5 mg L^−1^, [H_2_O_2_] = 146 mg L^−1^.

Taking this into account, an inhibition of photo-Fenton in the presence of chlorides can be expected. For instance, sulfamethoxazole (50 mg L^−1^) degradation was studied in deionized water and in real marine water from the Mediterranean Sea using 2.6 mg L^−1^ of ferrous salts in solution with different concentration of hydrogen peroxide at pH 2.8. Almost 90% of mineralization was achieved in deionized water, while in seawater, the process was clearly slower and stopped at near 45% mineralization [46]. However, as can be seen in Figure 2A, this effect is also due to the presence of other anions or compounds present in seawater and not only due to chlorides, as the inhibitory effect observed was lower when working in distilled water with the addition of 30 g L^−1^ NaCl (high salty water, HSW), simulating seawater salinity [18].

Nevertheless, the extent of inhibition due to chlorides is strongly pH-dependent, as the coordination of Fe(III) depends on time on the pH of the medium [47]. The chlorinated complexes are predominating at low pH values and working at higher pHs (e.g., pH = 3.5) has been proposed as a strategy to minimise these interferences [43,48]. Interestingly, the presence of moderate concentrations of chlorides (1 g L^−1^ of NaCl) has been demonstrated to accelerate the removal of a mixture of pollutants in a solar-simulated process driven at pH = 5; however, at higher concentrations (30 g L^−1^ of NaCl), the effect was again detrimental (Figure 2B) [18].

Another way to overcome the effect of chlorides is adding ligands able to form Fenton-like active complexes that displace Cl^−^ from the coordination sphere of iron. This strategy has been successfully followed to treat a mixture of pollutants at pH = 5 using catechol as a chelating agent in saline environments [47,49].

At this point, it is important to distinguish the interference caused by the presence of iron-chloride complexes and that caused by chloride radical scavenging. Monitoring the decomposition rates of hydrogen peroxide can be useful for this purpose [18]. If the scavenging of ^•^OH by chlorides is the major mechanism, then peroxide consumption would not be affected, but the degradation of pollutants would be lower since the ^●^OH would be interacting with the Cl^−^. On the other hand, lower H_2_O_2_ consumption recorded in the presence of chlorides seems to point iron to the decreased activity of Fe(III) because of chlorides’ complexation as the major cause for Fenton inhibition, rather than the quenching of ·OH by chlorides.

Finally, it should be highlighted that chloride radicals (as well as other halogenated radicals such as Br^●^, vide infra) can also lead to the formation of halogenated by-products [50,51], a matter of concern due to the potentially carcinogenic effects and the higher toxicity of this kind of compounds. Also, Cl^●^ can act by H-subtraction rather than addition (generating hydroxylated products).

### 2.2. Bromides

Although Br^−^ is usually present in negligible concentrations in natural water, this is not always the case in highly saline waters, such as seawater or hydraulic fracturing wastewater, where its concentration is remarkable (ca. 60–80 mg L^−1^) [52], and therefore the formation of halogen-radicals must be borne in mind [53].

Bromide (Br^−^) can react with ·OH, with a very high kinetic rate constant (above 10^10^ M^−1^ s^−1^), acting as an ·OH radical scavenger [54,55]; with the additional inconvenience of the formed Br^●^ leading to hypobromite (HBrO/BrO^−^), which is eventually further oxidised until the formation of bromate (BrO_3_^−^), a carcinogenic compound [56].

### 2.3. Fluorides

The presence of fluorides in water is commonly very low, and their effect is commonly neglected. For instance, in seawater, the concentration is reported to be 1 mg L^−1^ [57]. However, it can be observed in Table 2 that the stability constant of the Fe(III) complexes with fluorides is several orders of magnitude higher than that of chlorides. Considering that the amount of iron added for mild photo-Fenton process is also commonly below 5 mg L^−1^, fluorides can inhibit the process by decreasing iron availability. This could be one of the causes that explain the lower performance of photo-Fenton in real seawater when compared with synthetic matrices consisting of 30–40 g L^−1^ of NaCl.

It is interesting to remark that the reaction of ·OH with F- is not favoured, thus disregarding the scavenging of hydroxyl radicals as the predominant mechanism.

### 2.4. Sulphates

Sulphates are able to form weak complexes with iron salts, such as FeSO_4_^+^ (log K = 2.12) [36], which can be relevant at some pH values. For instance, at pH 3, Fe(OH)^2+^ and FeSO_4_^+^ are the major ionic ferric species. Under UV irradiation, FeSO_4_^+^ produces ferrous ions and sulphate radical anions, SO_4_^·−^, although the quantum yield for its photolysis is low when compared with Fe(OH)^2+^ [48]. Furthermore, sulphates act as radical scavengers, according to Equation (11). The sulphate radicals have a high redox potential, 2.5 V [58]; in fact, the use of these radicals for wastewater treatment has been receiving increasing attention in the last 15 years [59].

HSO_4_^−^ + HO^●^ → SO_4_^−●^ + H_2_O (11)

The effect of sulphates in the photo-Fenton process sometimes remains hidden since most of the authors use the iron(II) sulphate heptahydrate, Fe(SO_4_)·7H_2_O, as a source of iron and sulfuric acid for adjusting the pH values.

### 2.5. Carbonates or Bicarbonates

Carbonates are commonly found in natural water, and their effect on photo-Fenton is, after chlorides, the one that has deserved more interest from researchers. While the speciation of other anions is not relevant, in the case of carbonates and bicarbonates it could be important for their interaction with iron. Their concentration depends on pH, and at pH < 5 their presence is negligible, as the formation of CO_2_ is favoured. On the contrary, at circumneutral pH (in the range of 6–9), bicarbonate is the predominant specie. At these pH conditions, the interaction between carbonate/bicarbonate and Fe(II)/Fe(III) is very complex and induces changes in the coordination sphere of ions that might affect the rate of hydrogen peroxide consumption, as described for chlorides. However, this fact is not necessarily detrimental for pollutants’ degradation, most probably due to the release of alternative oxidation species [60].

Carbonates are able to react with hydroxyl radicals to form carbonate radicals (Table 1), which have lower redox potential (1.78 V) when compared with ·OH and hence become more selective [61]. Thus, the substitution of ·OH by ·CO_3_^−^ modifies the oxidation rates of pollutants and systematically results in lower mineralization.

The buffer effect that carbonates provide to the solution can also play an important role. At pHs close to neutrality, a decrease in the pH during the treatment is commonly observed, caused either by the acidic properties of Fe(III), or by the formation of carboxylic acids in the oxidation of organics, approaching the optimum value for photo-Fenton, and thus accelerating the process. However, when carbonates are present, the solution is buffered and the pH decrease is not remarkable. In order to avoid this effect, consecutive additions of iron have been proposed until the pH stabilisation of carbonates is suppressed and pH decreases [62].

### 2.6. Borates

The effect of borates on Fenton process has not been studied in-depth because of the low concentrations they reach in natural systems. Despite the iron-borate complex having a significant stability constant value (logβFe[B(OH)_4_]^2+^ = 6 [39]), the low concentration of B(OH)_4_^−^ results in a minor role in iron complexation in seawater or in other relevant water matrices. Borates also have negligible bimolecular rate constants towards HO^●^; thus, the scavenging role of ·OH is not relevant.

However, in some studies, a borate buffer is employed, and at these concentrations iron complexation cannot be neglected. For instance, it was observed that the performance of photo-Fenton was worse when a borate buffer was used, with lower H_2_O_2_ consumption [63]. In contrast, it was observed that the presence of a borate buffer has no effect on the UV/H_2_O_2_ process; therefore, the formation of a Fenton-like inactive complex at high borate concentration may explain this behaviour.

### 2.7. Phosphates

Although phosphates can chelate Fe^2+^, it does not seriously decrease the reaction rate from the Fenton reaction. The reason why they significantly affect photo-Fenton reactions is because Fe^3+^-phosphate complexes easily precipitate even at acidic conditions.

The interference caused by the presence of phosphates on photo-Fenton processes is among the most undesirable, since it completely inhibits the catalytic role of ion [64]. This fact is attributed to the formation of a non-soluble and non-active iron phosphate, FePO_4_, with a value of K_ps_ of 9.91·10^−16^ [15]. In consequence, when phosphates are present in an effluent, they have to be removed before Fenton and related processes can be applied. In consequence, phosphate-based buffers should be avoided in photo-Fenton experiments at controlled pH.

Despite phosphates not commonly being present at noticeable concentrations in relevant water matrices, they can be released as a result of the degradation of organophosphate compounds, such as pesticides. In these cases, an addition of extra amounts of iron to replace that inactivated by phosphates is needed [65]. Furthermore, it should be noted that in many cases, a phosphate buffer is used for experiments related to the photo-Fenton process at near-neutral pH, so the results obtained in these cases could be erroneous and inaccurate.

### 2.8. Nitrates

The presence of nitrates has been reported to exhibit no significant effect on Fenton chemistry, and hence, this process is efficient even at high concentrations of this anion [66]. Despite nitrates being known to generate hydroxyl radicals under UVA irradiation (Equation (12)), the quantum yield of this process is very low (2·10^−3^ mol of HO^●^ per mol of photons absorbed) and the ·OH formed through this way is negligible vs. that formed by the photo-Fenton process [67]. Also, other radicals can be photogenerated from nitrates (NO^●^, NO_2_^●^ and ONOO^●^), but this subject falls beyond the aim of this work [68]. Finally, HO^●^ scavenging by nitrates is negligible.

NO_3_^−^ + H_2_O + hν → HO· + NO_2_· + HO^−^ (12)

In summary (Table 3), inorganic anions can alter photo-Fenton efficiency through two dominant mechanisms: (i) by modifying iron speciation, changing the Fe(II)/Fe(III) redox cycling rate and affecting light absorption and photoreduction efficiency; (ii) by scavenging hydroxyl radicals, forming less reactive or more selective species such as Cl_2_•^−^, CO_3_•^−^, or SO_4_•^−^. While chlorides and carbonates may act as inhibitors at acidic pH, they can promote reactivity under near-neutral conditions through changes in iron coordination. Phosphates, in contrast, form insoluble FePO_4_ and completely suppress catalytic activity.

## 3. Applications for Pollutants’ Abatement

Although numerous studies have demonstrated the applicability of Fenton and photo-Fenton systems to saline effluents, several operational limitations remain. High concentrations of chlorides and carbonates can slow mineralisation, while produced water and textile brines often contain metals or surfactants that interfere with iron redox cycling. Moreover, scaling up these treatments requires addressing issues such as excessive reagent consumption, iron sludge generation, poor light penetration, and the need for post-treatment steps.

Chloride is one of the highest concentrations in waters to be treated before a wastewater treatment plant (pre-treatment) or after (tertiary treatment) [69,70]. In addition, it is the most abundant anion in seawater (water near ports or refineries, ballast waters, marine fish farms), but also in industrial effluents (textile or petrochemical industries) or membrane retentates; in some cases, Fenton-based processes have been studied as a treatment option for these waters. In most cases, chlorides play a major role, and they are found at concentrations well above 1 g L^−1^. Perhaps the type of saline water most widely treated by photo-Fenton process is produced water (PW), together with effluents from the textile industry.

PW refers to water obtained in the petrochemical or natural gas industry due to fossil fuel extraction activities [71]. This type of effluent presents a high concentration of organic and inorganic species, which are dragged from subsoil. Typically, this water contains between 35 and 240 g L^−1^ of NaCl and total organic carbon (TOC) of around 500 mg C·L^−1^ [72,73].

Nowadays, PW treatment is based on physical separation technologies, methods that do not allow obtaining water with compatible standards for reuse [74]. For that, AOPs are being widely studied due to their effectiveness in the oxidation of organic compounds and their good combination with different treatments [71,75]. Table 4 summarises the information on the application of photo-Fenton for PW. In general, it is applied in combination with other processes; the most widely employed are flotation/sedimentation and membranes.

Lui et al. [76] applied the Fenton and UV–Fenton processes for the TOC removal of a real shale flowback water, with mineralisations near 35% and 60%, respectively, after 90 min of treatment. High concentrations of chloride ions in the matrix form complexes with Fe^2+^/Fe^3+^, reducing their reactivity with H_2_O_2_. During UV-assisted Fenton oxidation, the reduction of Fe(III) to Fe(II) is accelerated, while the generation of ∙OH radicals enhances TOC removal. This occurs because to the photolysis of Fe(III) complexes (such as FeCl_2_^+^) into Fe^2+^ and Cl^−^ further increases the reduction of iron.

Sinha et al. [77] applied different conditions of the Fenton process to search for optimum conditions and to obtain detailed kinetic models of the reactions. Interestingly, results show similar values of mineralisation between pH 3 and 7, around 60–75% of TOC removal; so, pH does not need to be adjusted. Also, the wastewater was pretreated before the application of the Fenton process. Nevertheless, the filtration with sand or the coagulation process applied do not reduce the amount of chlorides [76,77].

Secondly, the use of membranes for the treatment of PW is an interesting option for the purification of this type of water [78]. However, the high concentration of organic matter can foul and wet the membrane, as well as worsen the quality of the distillate obtained [72]. For that, Fenton processes are applied as a previous step to remove these compounds before the use of membranes, both to reduce the organics in the membrane distillation effluent and to obtain better wastewater values in terms of toxicity. Using a synthetic effluent based on real wastewater, different works were performed. Ricceri et al. [73] studied the effect of the Fenton experiments, adding H_2_O_2_ at different times. This combination improves the flux of membrane distillation. They affirm that the Fenton remained effective despite the high chloride concentration in the feed solution. So, that makes it suitable for applying the process in complex hypersaline environments.

In other work, the effect of Fenton and modified Fenton (with iron ligands) processes was checked. Chloride ions pose a significant challenge to the efficacy of the Fenton reaction in hypersaline-produced water, though humic acids may support Fe(III)-Fe(II) recycling. Despite this, the observed TOC removal rates and degradation yields highlight the Fenton reaction’s potential for effective decontamination even in the presence of high chloride concentrations. The use of the Fenton removes organics in the membrane effluents and, in combination with the membrane processes, provides a better effluent in terms of toxicity. Interestingly, in spite of modified Fenton reaching better results in terms of pollutant degradation, it does not present beneficial effects on the quality and productivity of the effluent [72].

**Table 4:** Application of Fenton-type processes for the treatment of produced water in the extraction processes of the petrochemical or natural gas industry (references given in the last column).

Parameter or Model Pollutant:	Industry	Salinity Level	Operational Parameters	Reference
*TOC*	Hydraulic fracturing	Real shale flowback water pretreated with coagulation [Cl^−^] = 8334 mg L^−1^	Fenton and UV–Fenton pH: 3–5 Iron source: Fe(II)–FeSO_4_·7H_2_O H_2_O_2_/Fe^2+^ = 110–150 H_2_O_2_/COD: 9–13 Solar irradiation: mercury lamp	[76]
*TOC*	Hydraulic fracturing	Flowback water filtered	Fenton pH: 2–12 Iron source: Fe(II)–FeSO_4_·7H_2_O [Fe^2+^] = 0.007–0.036 M H_2_O_2_: 0.5–1.9 M	[77]
*Xylenes, benzene, toluene, and methyl tert-butyl ether (MTBE), Cyclohexane, phenol*	Petrochemical	Synthetic water (ppm): NaCl: 100,000 CaCl_2_: 2500 MgCl_2_: 4000	Fenton pH: 3, 4 with EDDS, 5 with citrate Iron source: Fe(II)–FeSO_4_·7H_2_O at 0.5–5 mM (Fe-EDDS and Fe-sodium citrate) H_2_O_2_: 5–100 mM	[72]
*P-xylene, benzene, toluene, and MTBE, SDS, cyclehexane, naphthalene, phenol*	Petrochemical	Synthetic water NaCl: 98.87 ppm	Fenton pH: 3 Iron source: Fe(II)–FeSO_4_·7H_2_O at 19 mM H_2_O_2_: 32 mM (in six additions)	[73]
*Toluene, xylene, naphthalene, phenol, acetic and malonic acids* *TOC removal*	Petrochemical	Seawater matrix	Fenton, photo-Fenton and sono-Fenton pH: 3 Iron source: Fe(II)–FeSO_4_·7H_2_O H_2_O_2_/Fe^2+^: (0.25–10) H_2_O_2_: H_2_O_2_/COD (2.1–10) Solar irradiation: UVC and UVA	[79]
*Chemical Oxygen Demand (COD), TOC and colour removal*	Natural gas field	Raw PW (southwest of Chongqing City, China)	Fenton, UV–Fenton and US–Fenton pH: 2–7 Iron source: Fe(II)–FeSO_4_·7H_2_O [H_2_O_2_]/[Fe^2+^] molar ratio (5–30) H_2_O_2_: H_2_O_2_/COD (1–10) Solar irradiation: Solarbox 3000e	[80]
*TOC*	Petrochemical	Synthetic effluent with phenol and real effluent of oilfield produced water	Photo-Fenton pH: 3 Iron source: Fe(II)–FeSO_4_·7H_2_O [Fe^2+^] = 0.75–3 mM H_2_O_2_: Dosage every 30 min 50–107.7 mM Solar irradiation: Solar light and simulated UVA light	[81]
*Oil and grease* *Soluble compounds as phenol*	Petrochemical	Simulated Produced Water: filtered seawater	Fenton and photo-Fenton pH: 3 Iron source: Fe(II)–FeSO_4_·7H_2_O at 133–300 mg L^−1^ H_2_O_2_: 600–1660 mg L^−1^ Solar irradiation: Solarbox 3000e	[74]
*Dissolved Organic Carbon (DOC)*	Hydraulic fracturing	Flowback water filtered [Cl^−^] = 13,600 mg L^−1^	Photo-Fenton pH: 3 Iron source: Fe(II)–FeSO_4_·7H_2_O [Fe^2+^] = 80 mg L^−1^ H_2_O_2_: 500 mg L^−1^	[82]
*Total of oils and grease*	Petrochemical	Aqueous saline solutions with crude oil [NaCl] = 200–4229 ppm	Photo-Fenton pH: 3 Iron source: Fe(II)–FeSO_4_·7H_2_O [Fe^2+^] = 0.1–44 mM H_2_O_2_: 10–44 mM Solar irradiation: 400 W Mercury vapour lamp	[83]
*COD*	Petrochemical	Oilfield fracturing wastewater	Fenton pH: 3 Iron source: Fe(II)–FeSO_4_·7H_2_O at [H_2_O_2_]/[FeSO_4_] ratio of 2 H_2_O_2_: 2 mM	[84]
*Hydrocarbons* *TOC*	Petrochemical	Aqueous saline solutions with raw gasoline [NaCl] = 200–2000 ppm	Photo-Fenton pH: 3 Iron source: Fe(II)–FeSO_4_·7H_2_O [Fe^2+^] = 0.5–1 mM H_2_O_2_: 100–200 mM Solar irradiation: Hannovia 450 W medium-pressure mercury vapour lamp	[85]

Likewise, another of the most polluting and wastewater-producing industries with a difficult effluent to treat is the textile industry. The high quantity of wastewater produced by this industry [86], together with the number of organic pollutants that are non-biodegradable and cannot currently be treated by conventional methods [87], creates the need to search for different methods for their treatment, such as AOPs. In addition, this type of wastewater has high mineral contents such as chlorides and also different toxic compounds, which makes its treatment even more difficult [88,89]. Generally speaking, it contains a large amount of different organic pollutants, low biodegradability, the presence of salts and colourants, and its value of kg of COD/ton of finished textile is around 115–175 kg COD/ton [90,91]. This is why research into applying new treatments focusing on advanced oxidation processes (such as Fenton or photo-Fenton) to remove organic compounds and dyes is gaining interest [92].

Table 5 shows different treatments performed to remove dyes and organic compounds from textile industry wastewater. The combination of different steps of coagulation-flocculation, followed by a Fenton-neutralisation process, was studied for this purpose. Different works report their application on real industrial textile wastewater, with a total decolouration of the water and a decreasing global COD and TOC of 89% and 87%, respectively. With this procedure, the wastewater treated complies with the current environmental regulations in Colombia [93,94]. In addition, granular heterogeneous Fenton has been investigated for removing azo dyes in seawater. In this study, the elimination of non-biodegradable organic contaminants was followed, using Direct Blue 17 as an example [92].

Another interesting method for this type of wastewater, as in the case of produced water, is the combination of membrane steps with the photo-Fenton process. Lebron et al. studied the application of the process in two cases: (i) for application in the concentrate water after the nanofiltration process, or (ii) as an intermediate step between microfiltration and nanofiltration to reduce flux problems due to organic matter deposition on the membrane. For this test, a real textile effluent was used. The work concluded that the best combination in terms of cost-effectiveness and the reutilization of the effluent in the process was applying the AOP after the nano-filtration step. Also, a process to remove iron should be a good combination to accomplish the requirements to reuse the water treated in more noble processes [95].

**Table 5:** Application of Fenton-type processes for the treatment of saline water from the textile industry. (References given in the last column).

Parameter or Model Pollutant:	Industry	Salinity Level	Operational Parameters	Reference
*Remazol Black, Perigen LDR*, *Synozol Yellow KHL (Reactive Yellow 145)*, *and Synozol Blue KBR (Reactive Blue 221)*	Textile industry	Conductivity, mS/cm: 36.9 (RB5 aqueous solution), 57.6 (industrial wastewater)	Fenton and electro-Fenton pH: 3 Iron source: Fe(II)–FeSO_4_·7H_2_O 0.35, 0.52, 0.87 g H_2_O_2_: 5–15 mM	[88]
*TOC*	Textile industry	Pretreated real textile wastewater	Photo-Fenton-like pH: 3–10 Iron source: BiFeO_3_—2 g L^−1^ H_2_O_2_: 10 mM Light source: simulated visible and UV-C light	[96]
*COD and colour*	Textile industry	Real textile wastewater pretreated by coagulation-flocculation	Fenton and photo-Fenton pH: 2.31 Iron source: FeSO_4_·7H_2_O [Fe^2+^]_0_/[H_2_O_2_]_0_ = 0.537 H_2_O_2_: [COD]_0_/[H_2_O_2_]_0_ = 0.374	[93]
*Azo dye–Direct Blue 17*	Textile industry	Synthetic seawater	Heterogeneous Fenton pH: 3 Iron source: granulated Fe_3_O_4_/Cellulose nanocomposite 1, 3 and 5 mg L^−1^ H_2_O_2_: 3.5 g (30% r.w.)	[92]
*COD = 712.8 mg* *L^−1^*	Textile industry	Industrial textile ‘SITEX’ wastewater Conductivity, mS/cm: 8.6	Electro-Fenton pH: 3 Iron source: iron-iron electrode Surface area: 28 cm^2^ Gap between electrodes: 1 cm	[97]
*COD*	Textile industry	Textile wastewater	Fenton pH: 3–5 Iron source: Fe:H_2_O_2_: 1:1–1:8.175 H_2_O_2_: C:H_2_O_2_: 1:1.125–1:1.38	[95]
*Azo dye*–*AY36*	Textile industry		Heterogeneous Fenton pH: 3 Iron source: pyrite 1 g L^−1^ H_2_O_2_: 2 mM	[87]
*Colour*, *turbidity and DOC*	Textile industry	Real textile wastewater pretreated by coagulation-flocculation	Fenton pH: 6 and 7 Iron source: FeSO_4_·7H_2_O [Fe^2+^]_0_/[H_2_O_2_]_0_ = 0.093 H_2_O_2_: [COD]_0_/[H_2_O_2_]_0_ = 3.51	[98]
*TOC*	Textile industry	Real textile wastewater pretreated by coagulation-flocculation	Fenton and photo-Fenton pH: 3–6 Iron source: FeSO_4_·7H_2_O Fe(II)—0.25–1.25 mM H_2_O_2_: 9.8–98 mM Light source: UV radiation	[94]

Besides their application in the petrochemical and textile industry, the combination of Fenton processes and membrane technology, among other additional steps, has been studied in different industries and applications (Table 6). Fenton is also being studied for its implementation in desalination plants. Due to the concept of zero discharge, the application of Fenton-like processes in cleaning waters from the membrane process could be useful to reuse it in the main process or to be applied for irrigation [99]. This AOP could be used as a previous step to eliminate some organics present in the seawater, improving the reverse osmosis process and the quality of the retentate, and also to treat the effluent derived from the alkaline cleaning of the membrane, which produced an important amount of wastewater [100].

Likewise, other industries produce wastewater with high amounts of chlorides and the presence of organic compounds that are difficult to treat. For example, the combination of Fenton-type processes and membrane technology is used to treat olive mill wastewater. This type of wastewater presents Cl^−^ values above 1 g L^−1^ and different organic pollutants, such as phenols [101]. Also, the development and optimisation of a pilot-scale continuous reactor were studied for this purpose [102].

Similarly, due to the treatment of actual urban wastewater with nanofiltration membranes, the secondary effluents obtained after the treatment have high salinity values ([Cl^−^] = 1.1–2.2 g L^−1^). Thus, electro-Fenton and solar photoelectro-Fenton were used to degrade organic microcontaminants under these conditions [103]. As a complement, studies have also been carried out to observe the effect of iron salts and hydrogen peroxide on polyamide membranes, as well as the quantities of these reagents that they can withstand [104].

And finally, as another example of possible application, due to the presence of herbicides in aquaculture areas, their removal from both river and marine waters is necessary, as they seriously affect natural ecosystems. Thus, solar-enhanced bio-electro-Fenton driven by sediment microbial fuel cell has been studied for their application in situ in seawater [105].

Hybrid approaches combining membrane filtration and photo-Fenton processes are increasingly proposed for saline matrices. Photo-Fenton pre-oxidation can reduce organic load, mitigate membrane fouling and improve flux recovery, while membrane concentration can enhance the efficiency of downstream oxidation by reducing the treatment volume. However, inorganic ions influence both steps: chlorides and sulphates affect radical formation, while high ionic strength accelerates fouling and scaling in membranes. Recent studies suggest that optimised hybrid configurations—such as pre-Fenton oxidation followed by nanofiltration or forward osmosis—can improve energy efficiency and effluent quality in produced water, textile brines and desalination concentrates. These hybrid configurations have been successfully explored both at lab and pilot scale, as illustrated by recent studies on photo-Fenton–membrane systems for wastewater treatment and Fenton pre-treatment prior to membrane distillation of produced water [106].

**Table 6:** Application of Fenton-type processes for the treatment of saline waters produced or used in different industries (references given in the last column).

Parameter or Model Pollutant:	Industry	Salinity Level	Operational Parameters	Reference
*Anionic modified potato starch from Cargill (C flake 35,704)*	Desalination plants	Synthetic effluent	Fenton pH: 3 Iron source: Fe(II)–FeSO_4_·7H_2_O from 50 to 16,560 mg L^−1^ H_2_O_2_: 800 to 8280 mg L^−1^	[99]
*Anionic modified potato starch from Cargill (C flake 35,704)*	Desalination plants	Synthetic effluent	Photo-Fenton pH: 3 Iron source: Fe(II)–FeSO_4_·7H_2_O from 40 to 1000 mg L^−1^ H_2_O_2_: 800 to 2000 mg L^−1^ Solar irradiation: - 2500 W Xenon lamp. Irradiance: 250 W m^−2^ - 4 × 15 W UVA lamps (300–400 nm)	[100]
*Phenolic compounds*	Olive mil wastewater	Olive mill wastewater Cl^−^: 1096.6 mg L^−1^	Photo-Fenton pH: 3.2 Iron source: Fe(III)–FeCl_3_⋅6H_2_O [FeCl_3_]/[H_2_O_2_] ratio = 0.01–0.04 *w*/*w* Total iron: 50.8 mg L^−1^ H_2_O_2_: 5% *w*/*v*	[101]
*Phenolic compounds*	Olive mill wastewater		Fenton pH: 3–7 Iron source: Fe(III)–FeCl_3_⋅6H_2_O FeCl_3_: 0.04–0.2 g/dm^3^ and [FeCl_3_]/[H_2_O_2_]: 0.006 to 0.26 *w*/*w* H_2_O_2_: 20 to 30 g/dm^3^	[102]
*Pentachlorophenol, terbutryn, chlorphenvinfos, and diclofenac*	Urban wastewater	Synthetic recipe of simulated nanofiltration retentate [Cl^−^] = 1100–2000 mg L^−1^	Electro-Fenton and solar photoelectron-Fenton pH: 8–8.6 Iron source: Fe_2_(SO_4_)_3_.×H_2_O Fe^3+^-EDDS: concentration rate of 0.1:0.2 mM	[103]
*Ametryn*	Aquaculture	Simulated seawater Salinity: 31%	Solar-enhanced bio-electro-Fenton pH: 7.99 Iron source: γ-FeOOH	[104]

## 4. Applications for Disinfection

As it has been mentioned in previous sections, Fenton and photo-Fenton processes are recognised for their potential in treating various types of wastewater, including marine waters, that can degrade organic pollutants but also inactivate different type of microorganisms (Table 7). Despite the effectiveness of the Fenton process in disinfection, limited studies have explored its application in seawater. Blanchon et al. [106] review the potential of heterogeneous photo-oxidation for microbial inactivation in seawater, highlighting that inorganic ions can have both scavenging and/or iron complexation effects in disinfection processes, which implies a dual role of an inhibitory effect or promoting effect, as discussed in previous sections. Thus, this suggests promising applications in aquaculture and marine industries, calling for further research to optimise and scale the technology for practical use. Gandhi & Prakash [107] also provide a comprehensive review of photo-disinfection processes for bacterial inactivation, highlighting the influence of water constituents such as dissolved organic matter, turbidity, and inorganic ions on process efficiency, but at lower concentrations than those naturally occurring in high-salinity environments.

The high salinity and typically high pH of marine waters can indeed affect the efficacy of Fenton processes. Rubio et al. [108] investigated the inactivation of *Escherichia coli* in artificial seawater using a photo-Fenton process with two different sources of UV (solar light and UV-C low-pressure lamp). When a UV-C source is employed, similar results were obtained either with or without the presence of natural organic matter (NOM) and the iron source (Fe^2+^, Fe^3+^). An improvement on kinetic rate constant was obtained, ranging 172–229% with respect to the single UV process, in this case being slightly higher than other aqueous matrices with less salinity. It agrees with the hypothesis presented in previous sections and the formation of complex among chlorides and iron. A UV-C source with a more energetic wavelength would easily photolyze the complex, although authors attained similar results to the pH of the solution (pH = 7.81) and iron precipitation. A similar approach was considered by Aguilar et al. [109], who, using a UV-C source, achieved optimal results with 10 mg H_2_O_2_ L^−1^ and 2 mg Fe^3+^ L^−1^, leading to a 44.4% and 50% increase in the disinfection rate constant of *Klebsiella pneumoniae* compared to UV/H_2_O_2_ or single UV-C radiation, respectively. This enhancement in disinfection efficiency was attributed to a mechanism involving cell adsorption and the formation of complexes between Fe^3+^ and Cl^−^.

In solar tests, the improvement obtained in the inactivation kinetic rate constant of *E. coli* was quantified as 252% and 159% with the presence or absence of NOM, respectively [108]. Accordingly, authors report a positive effect of dissolved organic matter in photo-Fenton systems in high-salinity aqueous matrices. In this study, some mentions of the formation of possible complex between iron and NOM are discussed, among the reasons of these improvements. However, there is no mention of the possible complex of iron and inorganics. In any case, although the improvement is clearly obtained, when it is compared with other water matrices, such as Milli-Q water or freshwater lake water, the inactivation rates are always faster than in seawater. On the other hand, Rommozzi et al. [110] explored the impact of various inorganic ions on the efficacy of the photo-Fenton process for the inactivation of *Escherichia coli* under solar irradiation (SODIS) and near-neutral pH. The presence of chloride ions (Cl^−^) enhances disinfection, attributed to the interaction between Cl^−^ and Fe^3+^, forming complexes with higher absorption coefficients and quantum yields for UV photolysis.

A modest increase in the inactivation rate was also observed with the presence of sulphate ions (SO_4_^2−^), as Fe^3+^ and SO_4_^2−^ could form FeSO_4_^+^, which may generate sulphate radicals (SO_4_^•−^) upon photolysis (see Section 2.4). Nitrate (NO_3_^−^) and nitrite (NO_2_^−^) ions enhanced disinfection kinetics at all tested concentrations, with nitrite having a more pronounced effect than nitrate. On the other hand, bicarbonate ions (HCO_3_^−^) enhance disinfection kinetics at low concentrations (10 mg L^−1^). However, at higher concentrations, they inhibit efficiency by acting as radical scavengers. Additionally, pH increases slightly as bicarbonate concentration decreases during the process. Their findings indicate that different salts can significantly alter the efficiency of the disinfection process; HCO_3_^−^ was found to slightly enhance inactivation kinetics under certain conditions, but at environmentally relevant concentrations, it acts antagonistically. Conversely, NO_3_^−^, NO_2_^−^, can benefit photo-Fenton disinfection. Photo-Fenton can also be faster with Cl^−^ and SO_4_^2−^, but increasing Cl^−^ may disrupt photo-Fenton, and increasing SO_4_^2−^ has the opposite effect. This aligns with the key points developed in Section 2.

**Table 7:** Application of Fenton-type processes for the treatment of micro-organisms present in different saline water matrices (references given in the last column).

Target Microorganism	Salinity Level	Operational Parameters	Reference
Faecal bacteria: *Escherichia coli*	Artificial seawater (35 g L^−1^ of sea salt) 50 mS/cm 19.49 g Cl^−^ L^−1^	Photo-Fenton pH: 7.81 Iron source: Fe(II)–FeSO_4_·7H_2_O at 1 mg L^−1^ H_2_O_2_: 10 mg L^−1^ Solar irradiation: 500 W/m^2^	[108]
Faecal bacteria: *Escherichia coli*	Milli-Q water + specific salts: 1–500 mg Cl^−^ L^−1^ 1–50 mg NO_3_^−^ L^−1^ 0.01–5 mg NO_2_^−^ L^−1^ 10–500 mg SO_4_^2−^ L^−1^ 5–100 mg HCO_3_^−^ L^−1^	Photo-Fenton pH: near-neutral. Iron source: Fe(II)–FeSO_4_·7H_2_O at 1 mg L^−1^ H_2_O_2_: 10 mg L^−1^ Solar irradiation: 620 W/m^2^	[109]
Faecal bacteria: *Escherichia coli*, *Klebsiella pneumoniae*	Artificial salty water 50 mS/cm 35 g NaCl L^−1^	Photo-Fenton pH: 7.3–8.5 Iron source: Fe(III)–FeCl_3_·6H_2_O at 2.6 mg L^−1^ H_2_O_2_: 10, 30 mg L^−1^ UV-C radiation: 16.7 μW/cm^2^	[111]
Marine bacteria: *Vibrio alginolyticus*	Seawater collected from Pearl River Estuary Salinity 28.8‰	Photo-Fenton pH: 6–8.1–10. Iron source: Fe(II)–FeSO_4_·7H_2_O at 0.9, 1.8, 2.7 mM H_2_O_2_: up to 48.9 μmol L^−1^ Visible Light (300 W xenon lamp): 60 mW/cm^2^	[112]
Marine bacteria and cyanobacteria: *Anabaena* sp. *Vibrio alginolyticus*	Ground saltwater Salinity = 35.8 PSU	Fenton pH: Iron source: Fe(II)–FeSO_4_·7H_2_O at 18 μmol: H_2_O_2_: 0.059, 0.118, 0.29 mmol	[113]
Marine phytoplankton: Diatoms, Green algae, Dinoflagellates, Prymnesiophytes	Artificial seawater Aquil	Fenton pH: Iron source: FeT 8.3 μmol (Fe-III 6.6·10^−13^ μmol. H_2_O_2_: 0, 0.8, 1.6, 3.2, 6.4 mg L^−1^	[114]
Marine phytoplankton: *Chlorella* sp.; *Dunaliella salina*	Seawater	Electro-Fenton pH: 6.2, 8.3 Fe (III) up to 1000 μg L^−1^ (pH 6.2) and 1500 μg L^−1^ (pH 8.3)	[115]

Other studies have collected real water samples and tested the applicability of Fenton-like processes. For instance, Wang et al. [112] demonstrated that a visible light-induced photo-Fenton system could achieve up to seven log-removal values of the marine bacterium *Vibrio alginolyticus* within 35 min of visible light exposure, by continuously generating H_2_O_2_ in situ via photocatalysis. Although this study did not comprehensively investigate the interaction of salts in the disinfection process, it suggests that Fenton-based processes can still be highly effective in marine environments when properly optimised. Conversely, Villar-Navarro et al. [116], while not testing Fenton processes, showed that the presence of small concentrations of iron (14.3 ± 4 µg L^−1^) does not provide additional disinfection in a solar/H_2_O_2_ system.

Fenton-based processes are also studied in the abatement of marine cyanobacteria and microalgae. The inactivation of algae in marine waters has been investigated using Fe-ACF (activated carbon fibre) electro-Fenton processes [115] and oxidants like H_2_O_2_ and S_2_O_8_^2−^ [113]. The electro-Fenton process demonstrated high efficiency in inactivating *Chlorella* and *Dunaliella* salina, achieving nearly 98% efficiency under neutral pH conditions. This process effectively combines indirect oxidation by •OH, direct oxidation by free chlorine, and coagulation and adsorption of iron hydroxide complexes, functioning well even at a pH of 8.3 [113]. In another study, H_2_O_2_ showed superior performance over S_2_O_8_^2−^ in inhibiting the growth of *Anabaena* sp., achieving over 90% inhibition within 72 h. However, in co-cultures with *Vibrio alginolyticus*, higher H_2_O_2_ concentrations were needed, and the presence of Fe^2+^ improved the inhibition by approximately 60% [113].

Microcosm studies explored the use of H_2_O_2_ with the total Fe present in natural seawater (8.3 µM) for controlling harmful brown tide algal blooms caused by *Aureococcus anophagefferens* [114]. This treatment effectively eradicated high-density blooms within 24 h without significantly affecting other phytoplankton species. The phytoplankton assemblage, particularly diatoms and green algae, showed only transient suppression and recovered within 72 h. Although cyanobacteria were not completely eradicated, their population was reduced by about 50% within 72 h.

Although with some particularities, the photo-Fenton process disinfection mechanisms can be similar on microalgae, cyanobacteria, and bacteria, influenced by intracellular and extracellular oxidative stress and the presence of various salts [108,109,112,115,116]. The Fenton-based process primarily involves extracellular generation of ^•^OH via reactions between H_2_O_2_ and ferrous ions Fe^2+^. These radicals attack cell walls and membranes, leading to structural damage. Intracellular mechanisms also play a role, where H_2_O_2_ can penetrate cells, reacting with intracellular iron to form ^•^OH, causing oxidative damage to vital cellular components [111,113]. The presence of salts such as chloride (Cl^−^) and sulphate (SO_4_^2−^) can further influence these processes [113] (Section 2). Chloride ions can reduce bacterial viability by affecting membrane integrity, especially in bacteria that are not adapted to high-salinity environments, such as faecal bacteria. If primary ^•^OH is generated outside, it can interact with chlorides and other ions and generate chloride-related radicals that may attack the membranes of the different microorganisms [110,111,113].

Bicarbonate can also scavenge hydroxyl radicals, forming less-reactive species and potentially diminishing the oxidative stress. On the other hand, nitrate and nitrite can enhance intracellular ^•^OH production [114]. The intracellular domain of cyanobacteria is particularly impacted by H_2_O_2_ due to cyanobacteria’s limited detoxification mechanisms [115]. Thus, H_2_O_2_ alone was effective in controlling certain photosynthetic microorganisms, but required precise dosing to avoid negative impacts on non-target marine organisms [115,116]. Overall, these interactions emphasise the complexity of Fenton and photo-Fenton processes, with distinct disinfection mechanisms, influenced by both extracellular and intracellular pathways and the presence of dissolved salts, which play critical roles in determining the overall efficacy of these processes.

## 5. Conclusions

In most cases, the negative effect on the based-Fenton processes due to inorganic ions present in complex matrices is caused by the inactivation of iron (as in the case of chlorides, phosphates or fluorides). These form complexes or inactive iron species, so that the regeneration of Fe^3+^ to Fe^2+^ is compromised, thus impeding the process and generation of ·OH. However, it should also be noted that the main effect of other anions (e.g., bromides) is derived from their scavenger effect on the hydroxyl radicals.

In terms of actual application, despite the fact that there is not a large number of studies focused on saline waters, the Fenton and photo-Fenton processes in this field have been gaining interest in recent times. As can be seen in the work carried out so far, Fenton remained effective despite the high chloride concentration in the water matrices. Moreover, it is efficient in cost-effective terms at the pre-industrial scale if it is compared to other AOPs. Nevertheless, it should be noted that in most cases the process is applied in combination with other methods, such as flotation/sedimentation, coagulation/flocculation and membranes; so, it is necessary to choose the processes and order of application depending on the salt matrix and the industrial effluent to be treated.

In addition, focusing on disinfection, while most studies conclude that Fenton-based processes are effective across different types of microorganisms, the required treatment conditions (e.g., oxidant doses, exposure times) can vary according to the type of microorganism and specific salinity. In summary, the application of Fenton and photo-Fenton processes in marine waters is feasible and can be effective when optimised for the specific water matrix and target microorganisms. The variability in results in different microorganisms points out the importance of tailored treatment strategies to achieve the desired disinfection outcomes.

To conclude, further studies in this field would be necessary to optimise the process in terms of time reduction, as well as in the application of organic substances that can act as chelating agents to (i) achieve pH values close to those of natural water and, in this way, try to reduce treatment costs; and (ii) counteract the iron deactivation provoked by the inorganic ions present on the highly saline matrices.

Overall, despite significant progress, several knowledge gaps and controversies persist in understanding the photo-Fenton performance under high-salinity conditions. Future research should address the following: (i) the quantitative contribution of alternative reactive species (e.g., Cl_2_•^−^, CO_3_•^−^) to pollutant degradation at circumneutral pH; (ii) the stability and recyclability of Fe–ligand complexes in saline matrices; and (iii) the potential coupling of photo-Fenton with biological or electrochemical post-treatments to improve overall process sustainability.

## Figures and Tables

**Table 3:** pH-dependent mechanisms of inorganic anions in photo-Fenton processes.

Anion	Complexation with Fe(III)/Fe(II)	Radical Scavenging
Cl^−^	Moderate complexation; active or partially active complexes. – More relevant near neutral pH (prevents Fe(III) precipitation). – Less influential at acidic pH.	Yes; reacts with HO^•^ → Cl^•^/Cl_2_^•−^. – More relevant at acidic pH.
SO_4_^2−^	Very weak complexation across all pH.	Yes, can form SO_4_^•−^ but not significant.
NO_3_^−^	Very weak complexation at all pH.	No significant scavenging.
HCO_3_^−^/CO_3_^2−^	Moderate, mostly inactive complexes at pH 5–8. – Reduced effect at acidic pH.	Yes; forms CO_3_^•−^. – Very relevant at neutral to alkaline pH.
PO_4_^3−^	Forms insoluble FePO_4_; strong inhibition. – Precipitation from pH > 3–4.	No; inhibition due to precipitation.
F^−^	Very strong complexation; inactive complexes across all pH.	No meaningful scavenging.

## Data Availability

No new data were created or analysed in this study. Data sharing is not applicable to this article.

## Abbreviations

The following abbreviations are used in this manuscript:

AOPs	Advanced Oxidation Processes
ROS	Reactive Oxygen Species
EDDS	Ethylenediamine-*N*,*N*′-Disuccinic Acid
UV	Ultraviolet
PW	Produced Water
TOC	Total Organic Carbon
COD	Chemical Oxygen Demand
DOC	Dissolved Organic Carbon
NOM	Natural Organic Matter
SODIS	Solar Water Disinfection
